# Effects of single family room architecture on parent–infant closeness and family centered care in neonatal environments—a single-center pre–post study

**DOI:** 10.1038/s41372-021-01137-z

**Published:** 2021-07-06

**Authors:** Emma Kainiemi, Pilvi Hongisto, Liisa Lehtonen, Bernd Pape, Anna Axelin

**Affiliations:** 1 Department of Nursing Science, University of Turku, Turku, Finland; 2 Faculty of Medicine, University of Turku, Turku, Finland; 3 Hospital District of Southwest Finland, Department of Pediatrics, Turku University Hospital, Turku, Finland; 4 Turku Clinical Research Center, University of Vaasa, Vaasa, Finland

**Keywords:** Paediatrics, Health care

## Abstract

**Objective:**

The aim of this study was to evaluate the effects of a single family room architecture in a neonatal intensive care unit (SFR-NICU) on parents’ presence, parent–infant skin-to-skin contact (SSC) and the quality of family centered care.

**Study design:**

Two cohorts of parents of preterm infants were compared: those in the unit before and after the move to SFR-NICU. The parents used daily diaries to report their presence and SSC, and they responded to daily text message questions about the quality of family centered care.

**Results:**

Parents spent more time in the SFR-NICU, but no significant change was found in SSC. Parents rated the quality of family centered care highly in both unit architectures, without a change in rating after the move.

**Conclusion:**

The SFR-NICU increased parents’ presence but not SSC. The change in architecture did not affect parents’ evaluations of the quality of family centered care, which was already highly rated before the move.

## Introduction

Caring for multiple infants in the same space has historically been the only option available for providing care for infants and their families in neonatal intensive care [1]. Open nurseries allow the staff to monitor several infants simultaneously [2–4] but offer only limited opportunities for parents’ presence and privacy. Over time, the importance of providing facilities that allow parents to participate as primary caregivers in a less stressful environment [4] has been recognized [1]. Therefore, NICUs are moving away from open layouts to units with single family rooms (SFR-NICUs) [5].

Previous studies have reported that the parents spend more time with their infants [6–8] and are more involved in infant care [2, 9] in SFR-NICUs. Their increased presence enhances the emotional bond between parents and infants [3], and the intimate atmosphere in SFR-NICUs [10] facilitates parent–infant interactions [7] and skin-to-skin contact (SSC) [2, 8, 9]. From the infant’s perspective, parents’ presence has been reported to decrease stress symptoms at term equivalent age [11, 12]. SFR-NICUs have been associated to better weight gain, lower mortality, lower rates of sepsis and bronchopulmonary dysplasia [6, 7, 13], better breastmilk production and success at breastfeeding [6, 8] and better cognitive development of very preterm infants [8]. The mechanisms behind these benefits are not known.

There is even less knowledge about the effects of SFR-NICU on parents’ experiences related to family centered care (FCC). While SFRs are beneficial for the infant–parent relationship, they might introduce challenges into parent–staff relationships. Earlier studies [8, 14, 15] have suggested that parents experience difficulties connecting and communicating with staff in SFR-NICUs and that they might feel less informed and less involved in decision making [15]. Parents might also experience loneliness [14] and isolation [7, 16], as it is more difficult to meet other parents [16] and get peer support in an SFR-NICU [15]. To evaluate the success of the SFR-NICU architecture, more information is needed on mothers’ and fathers’ experiences with FCC, including communication, shared decision-making, participation and emotional support.

For supporting the parent–infant closeness and providing care that is fulfilling the elements of FCC, a systematic change and high valuation towards FCC mindset is needed [17]. FCC is not only dependent on the environment and facilities but also on the care culture and practices adopted by the unit and the relationships the staff is able to establish with the parents [18]. We can gain valuable information about the role of SFR-NICU architecture on parents’ presence and experiences of FCC by comparing one unit with the same staff and similar competencies to provide FCC, before and after a move to SFR-NICU.

The aim of this study was to evaluate the effects of an SFR-NICU on the duration of parents’ presence and parent–infant SSC and on the parents’ experiences of the quality of FCC by comparing one NICU before and after a move to an SFR-NICU.

## Materials and methods

### Study design

This pre–post study was conducted in two study points before and after the unit changed architectural layout. No major changes were made into the care policies between the study points. The parents of preterm infants were asked to report their presence and the duration of SSC by filling out daily diaries for 2 weeks and to evaluate the quality of FCC by responding to daily text message questions throughout their hospital stay. The first time period was September 2013–March 2014, when the unit had shared patient rooms, and the second time period was April 2018–February 2019, after the NICU had moved to an SFR-NICU architecture.

### Setting and participants

The NICU of Turku University Hospital provides neonatal care from admission until discharge, including level III neonatal care. In 2013 and 2014, the unit had two to four patients in one room with no facilities for parents to stay overnight, and patient rooms offered only limited privacy for families. Parents’ presence and participation were not limited, and parents could visit the infant anytime. In addition, no restrictions were set for the visits of significant others, and the siblings, grandparents and friends could visit the family anytime. The unit was furnished with resting chairs, and although not kept permanently next to each infant crib or incubator, chairs or an adult bed were provided for SSC when needed, and parents were actively encouraged to initiate SSC with the infant. Before the study started, the whole team of healthcare professionals participated in Close Collaboration with Parents training, which was intended to update the care culture in the unit and to prepare the staff for SFR-NICU by increasing their capacity to support the early parent–infant relationship and to allow and provide support for the parents to be the primary caregivers for the infant [14, 19]. The unit moved to the SFR-NICU in April 2014.

In 2018–2019, the unit had 11 SFRs equipped with at least one parent bed, and another was provided if needed, allowing the parents to be present with their infant 24 h a day. One reclining chair was also kept in the room permanently, and another was provided if needed for twins. Neonatal care of all intensity levels was provided in the SFRs. The doors of SFRs were kept closed, and the staff had no visual contact with those in the rooms. In addition, the unit had three shared patient rooms for from two to four patients, which were used especially for short-term patients when all SFRs were occupied. Preterm infants had priority in the allocation of SFRs. Half of the SFRs had a private toilet and shower, while other parents used a toilet and shower on the corridor. The unit had a parents’ lounge and a kitchen for preparing food. The visitation rules regarding significant others remained unchanged. The characteristics of both unit environments are presented in Table 1.

**Table 1:** Unit characteristics in the unit with shared patient rooms and SFR-NICU.

	Unit with shared patient rooms	SFR-NICU
Admissions per year	638	508
Care days per month^a^	898	849
Doctors/weekday	4	4
Nursing staff/weekday	13	14
Sources of support	a, b, c, d	a, b, c, e
Couplet care	No	Sometimes
Any limitations yes/no	No	No
Significant others/visits	a, b, c, d	a, b, c, d
Facilities for the parents	a	a, b, c
Single family rooms	0	14

Sources of support: (a) psychological support by a psychologist or social worker or infant mental health nurse, (b) spiritual support by a pastor or chaplain or other religious professional, (c) social worker for social benefits and home care coordination, (d) nurse visits to home after discharge, (e) routine follow-up visits in hospital during the first month after discharge.

Facilities for the parents: (a) room for parents to relax and socialize with the other parents, (b) a shower dedicated for parents, (c) facilities for the parents to cook/warm up food, (d) routinely provide meals for the mother or father.

Significant others/visits: (a) siblings, (b) grandparents, (c) friends, (d) others.

^a^Care days per month during the study periods, infants receiving high intensive care multiplied by 3 and infants receiving intensive care by 2.

**Fig. 1:**
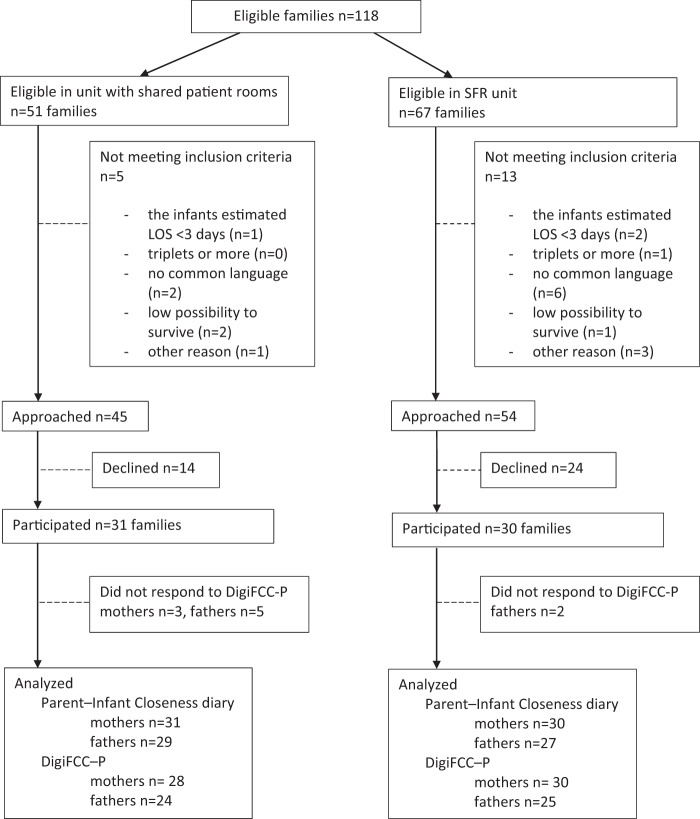
Enrollment of the study population in the unit with shared patient rooms and SRF-NICU described in a flow chart.

All the parents of preterm infants born before 35 weeks of gestation and admitted to the NICU were asked to participate in the study. Participation in the study had to begin within 6 days of birth. Only the parents of preterm infants were included because their expected length of hospitalization allowed collection of a sufficient amount of data. Families were excluded if (1) the expected duration of hospitalization was less than 3 days, (2) the infants were triplets, (3) the parents could not speak fluent Finnish or (4) the infant’s condition was critical and their survival uncertain. The target sample was 30 families for each time period. (Fig. 1.).

### Measurements

#### The parents’ presence and physical closeness

Parent–Infant Closeness Diary [20] was used to collect data on physical closeness between parents and infants. After recruitment, parents filled out diaries daily for 2 weeks or until discharge, if sooner. The diaries were in a paper format and they were kept in a folder in the patient rooms. One diary page represented 1 day in 5-min intervals. There were separate timelines for presence in the NICU and duration of SSC for both parents. Parents’ presence was defined as being inside the NICU. SSC was defined as the infant being held on the parent’s bare chest, wearing only a diaper and maybe a cap. An extra page was included for parents to explain the reasons for missing diary days.

#### The quality of FCC from the parents´ perspective

Parents’ experiences of the quality of FCC were assessed using the Digital Family Centered Care–Parent Version (DigiFCC–P) [20]. The DigiFCC–P includes one question on each of the following core elements of FCC: (1) active listening, (2) parent participation in infant care, (3) individualized parent education, (4) parent participation in decision-making, (5) parents´ trust in staff regarding infant care, (6) parents´ perception about the staff trusting them in infant care, (7) parents being involved in medical rounds, (8) individualized information and (9) emotional support. A secured website was used to send parents one of the nine questions in random order, using a short message service (SMS). One question was sent every evening until discharge or transfer to another unit. The parents used a 7-point Likert scale to answer the questions, with higher values representing more positive experiences. The value zero indicated that the parent was not in the unit that day. If a response was not received from the parent, the same question was sent as a reminder the next evening. No further SMSs were sent if the parent did not respond to the reminder. The parents of twins evaluated the mean of the quality of FCC for both twins.

The gestational age, birth weight, length and head circumstance, gender, delivery, singleton/twins and the length of hospitalization were identified as background factors for the infant. Parents’ background factors included age, education, socio-economic status, smoking, time of the first interaction with the infant, distance between home and hospital, relationship and the number of siblings at home. A unit characteristics survey was completed by the head neonatologist of the unit (LL) to provide information about the resources and practices in the unit at both time points.

Favorable ethical reviews were received from the Ethics Committee of the Hospital District of Southwest Finland (62/1802/2013, 08/011/18) and Turku University Hospital as a part of an international study. The research nurse responsible for recruiting the parents provided both written and oral information about the study and informed the parents about the opportunity to withdraw their participation at any time. The parents had time to consider participation overnight, and they signed informed written consent before participating in the study.

### Statistical analyses

We assessed the differences in background characteristics of families who agreed and who declined to participate and between the two NICU environments with Fisher’s exact test for categorical variables. For quantitative variables the Student-*t* was used for normally distributed variables and Wilcoxon rank sum test for nonparametric variables.

We calculated a monthly workload for staff using patient days in the unit and weighting them so that high-intensity care days were multiplied by three, medium-intensity care days by two and observation/monitoring days by one. We used Pearson’s correlation coefficient to assess the difference in the workload between two NICU environments and the association between the monthly workload and the parents’ experiences of the quality of FCC.

We compared median durations of presence and SSC between environments using analysis of covariance (ANCOVA), controlling for parent education, singletons/twins, relationship, siblings, and distance from home to hospital. We had to square root transform durations in order to obtain normally distributed residuals, implying that untransformed changes in duration become a function of reference duration. All changes of duration reported in the results section use median durations in shared patient rooms as reference duration.

ANCOVA was used to investigate differences in parents’ average evaluations of the quality of FCC, controlling for parent education, singletons/twins and relationship. We applied SPSS25 and SAS for Windows version 9.4 in the analyses.

## Results

### Parent and infant characteristics

Thirty-one families from the unit with shared patient rooms and 30 families from the SFR-NICU participated in the study. The participating families did not differ statistically significantly from those who declined participation in terms of infants’ gestational ages (*p* = 0.11) nor in distance from home to hospital (*p* = 0.56). Among participating families, the infants did not differ significantly in their background characteristics between the two study points. The only statistically significant difference was a lower educational level in the mothers (*p* = 0.006) and fathers (*p* = 0.004) in the unit with shared patient rooms as compared to the parents in the SFR-NICU. Infant and parent background characteristics are presented in more detail in Table 2.

**Table 2:** Infant and family characteristics in the unit with shared patient rooms and SFR-NICU.

Infant	Unit with shared patient rooms	SFR-NICU	*P* value
	*n* = 42	*n* = 40	
Gestational age (weeks) *Median (q1, q3)*	32.3 (29.8, 34.1)	32.0 (29.3, 34.0)	0.4112
Weight at birth (g) *Median (q1, q3)*	1770 (1195, 2121)	1560 (1256, 2042)	0.8166
Length at birth (cm) *Mean (sd)*	41.8 (3.6)	41.3 (4.5)	0.5854
Head circumstance at birth (cm) *Mean (sd)*	29.2 (2.8)	28.7 (3.5.)	0.4377
Gender *n* (%)			
Female	17 (40.5)	20 (50.0)	0.5058
Male	25 (59.5)	20 (50.0)	
Delivery *n* (%)	*n* = 41		0.3474
C-section	25 (61.0)	29 (72.5)	
Vaginal	16 (39.0)	11 (27.5)	
Twins n (%)			
Single baby	19 (45.2)	19 (47.5)	1.000
Twins	23 (54.8)	21 (52.5)	
Length of hospital stay (days) *Median (q1, q3)*	25 (13.8, 46)	35 (15.5, 60)	0.1784
Mothers and fathers	2013–2014	2018–2019	*P* value
	*n* = 31 families	*n* = 30 families	
	*n* = 31 mothers	*n* = 30 mothers	
	*n* = 29 fathers	*n* = 27 fathers	
Age (years)			
Mother *Mean (sd)*	30.8 (4.9)	32.8 (5.4)	0.1363
Father *Median (q1, q3)*	32.0 (30, 35.5)	33.0 (28.0, 36.0)	0.8454
Education *n (%)*			
Mother			
Primary education	0	0	0.0059
Second level	16 (57.1)	7 (25.0)	
Bachelors degree	10 (35.7)	10 (35.7)	
Masters/doctors degree	2 (7.1)	11 (39.3)	
Father			
Primary education	0	3 (5.6)	0.0041
Second level	17 (60.7)	9 (48.2)	
Bachelors degree	11 (39.3)	8 (30.8)	
Masters/doctors degree	0	6 (11.1)	
Occupation *n* (%)			
Mother	*n* = 30		
Paid work	18 (60.0)	22 (73.3)	0.2140
Unemployed	5 (16.7)	2 (6.7)	
Student	4 (13.3)	2 (6.7)	
Stay at home parent	1 (3.3)	4 (13.3)	
Other	2 (6.7)	0	
Father			
Paid work	26 (89.7)	25 (89.3)	1.000
Unemployed	2 (6.9)	2 (7.0)	
Student	0	1 (3.6)	
Stay at home parent	0	0	
Other	1 (3.5)	0	
Smoking *n* (%)	*n* = 30		
Mother	0	1 (3.3)	1.000
Father	8 (25.6)	4 (13.8)	0.3313
Relationship *n* (%)	*n* = 29		
Single	1 (3.5)	2 (6.7)	1.000
In relationship, not cohabiting	2 (6.9)	2 (6.7)	
In relationship, cohabiting	26 (89.7)	26 (86.7)	
Distance from home to hospital (km) *Median (q1, q3)*	18 (8, 100)	12 (5, 72)	0.4774
Home living children *n* (%)	*n* = 30		
No	13 (43.3)	15 (50.0)	0.7961
Yes	17 (56.7)	15 (50.0)	

*q1, q3* lower quartile, upper quartile, *sd* standard deviation.

### Parents’ presence and SSC in two NICU environments

Durations of parents’ presence and SSC are documented in Table 3. The time when either parent was present increased from a median of 5.9–10.2 h per day after the move from the unit with shared patient rooms to the SFR-NICU. The increase in the parents’ presence was similar during daytime and nighttime: parents’ daytime (8 a.m.–8 p.m.) presence increased by mean of 1.7 h and the nighttime (8 p.m.–8 a.m.) presence by mean of 1.7 h. Mothers’ presence increased from a median of 5.2 h to 9.1 h; fathers’ presence increased from a median of 3.6–5.9 h per day after the move. The adjusted statistical model indicated a 3.9h increase in the time either parent was present (*p* < 0.0001); a 3.5h increase in mothers’ presence (*p* < 0.0001); and a 2.3h increase in fathers’ presence (*p* = 0.0069).

**Table 3:** Parents’ presence and SSC in unit with shared patient rooms and in SFR-NICU.

Parent	Closeness, hours per day	Variables	Unit with shared patient rooms	SFR-NICU
Either parent	Presence	*N*	30	30
		Median (q1, q3)	5.9 (4.9; 7.6)**	10.2 (7.6; 13.2)**
		Min; Max	2.3; 10.5	4.0; 21.0
	SSC	*N*	30	30
		Median (q1, q3)	3.0 (2.3; 4.3)	4.1 (2.4; 5.5)
		Min; Max	0.3; 8.0	0; 7.8
Mother	Presence	*N*	30	30
		Median (q1, q3)	5.2 (4.6; 7.2)**	9.1 (7.2; 11.3)**
		Min; Max	1.8; 9.1	4.0; 17.4
	SSC	*N*	30	30
		Median (q1, q3)	2.0 (1.5; 2.7)	2.4 (1.3; 3.0)
		Min; Max	0.3; 5.1	0; 5.9
Father	Presence	*N*	28	26
		Median (q1, q3)	3.6 (2.6; 5.0)*	5.9 (3.4; 8.1)*
		Min; Max	0; 7.0	0.5; 13.4
	SSC	*N*	28	26
		Median (q1, q3)	1.0 (0.1; 1.8)	1.5 (0.6; 2.3)
		Min; Max	0; 3.2	0; 3.6

*q1, q3* lower quartile, upper quartile.

*p < 0.05, **p < 0.0001, ANCOVA controlling for parent education, singletons/twins, relationship, siblings and distance from home to hospital.

There were no statistically significant differences in the duration of SSC in the two NICU environments. The duration total SSC was a median of 3.0 h before the move and 4.1 h after the move to the SFR-NICU. Median duration of mother–infant SSC was 2.0 h per day before the move and 2.4 h per day after the move; median duration of father–infant SSC was 1.0 and 1.5 h, respectively.

Siblings at home decreased the mothers’ presence by 1.25 h (*p* = 0.04) and fathers´ presence by 1.49 h (*p* = 0.01). Mother–infant SSC was not affected by background factors. Father–infant SSC decreased by 0.12 h per week by the increase in gestational age (*p* = 0.02) and by 1.2 h if the infant was a twin (*p* = 0.002). The duration of total SSC per infant by either parent increased by 1.4 h if the infant was a twin (*p* = 0.04).

### Parental experiences of the quality of FCC in two NICU environments

A total of 1229 responses were received to the DigiFCC–P in the unit with shared patient rooms and 1641 in the SFR-NICU. Mothers gave 57.6% (shared patient rooms 668, SFR 985) and fathers 42.4% (shared patient rooms 561, SFR 656) of the answers.

The parents offered highly positive evaluations of the quality of FCC, regardless of the unit architecture. No statistically significant differences were found between the two NICU environments. The mean of mothers’ total scores was 5.97 ± 0.59 in the SFR-NICU and 6.15 ± 0.51 in the unit with shared patient rooms (*p* = 0.27, adjusted *p* = 0.19). The mean of fathers´ total score was 5.86 ± 0.61 and 5.73 ± 0.81 (*p* = 0.88, adjusted *p* = 0.33), respectively. Both parents rated participation in medical rounds, emotional support and participation in infants’ care as the weakest elements in both NICU environments (Table 4).

**Table 4:** Parents´ replies to DigiFCC–P in the unit with shared patient rooms and SFR-NICU.

DigiFCC–P question	Mothers			Fathers		
Mean ± s.d	Unit with shared patient rooms	SFR-NICU	*P* value	Unit with shared patient rooms	SFR-NICU	*P* value
1. To what extent did the staff listen to you today?	6.48 ± 0.63	6.57 ± 0.44	0.11	6.40 ± 0.90	6.11 ± 0.92	0.94
2. To what extent did you participate in your baby’s care today?	5.76 ± 1.40	5.67 ± 0.98	0.24	5.09 ± 1.35	5.18 ± 1.23	0.18
3. To what extent did the guidance provided by the staff meet your needs today?	6.01 ± 0.94	6.31 ± 0.87	0.09	6.41 ± 0.92	6.13 ± 0.91	0.45
4. To what extent was your opinion considered in decisions made about your baby today?	6.10 ± 1.04	6.21 ± 0.86	0.71	5.82 ± 1.06	5.88 ± 1.31	0.16
5. To what extent did you trust the staff in the care of your baby today?	6.66 ± 0.56	6.53 ± 0.69	0.51	6.70 ± 0.43	6.00 ± 1.10	0.16
6. To what extent did the staff trust you in the care of your baby today?	6.42 ± 0.76	6.48 ± 0.63	0.28	6.36 ± 0.95	6.35 ± 0.61	0.92
7. To what extent did you participate in discussions during the medical round?	4.63 ± 1.62	5.28 ± 1.64	0.12	3.81 ± 2.02	4.23 ± 1.83	0.89
8. To what extent did the information provided by the staff meet your needs today?	6.17 ± 1.10	6.27 ± 0.73	0.51	6.33 ± 0.72	6.32 ± 0.90	0.41
9. To what extent did the staff offer you emotional support today?	5.32 ± 1.58	5.52 ± 1.34	0.70	5.76 ± 1.36	5.03 ± 1.38	0.87
Total	5.97 ± 0.59	6.15 ± 0.51	0.19	5.86 ± 0.61	5.73 ± 0.81	0.33

The monthly workload during the study periods was a mean of 898.0 weighted patient days in the unit with shared patient rooms and 849.4 in the SFR-NICU. The difference was not statistically significant (*p* = 0.38). The monthly workload did not correlate with the parents’ experience of the quality of FCC (mothers *p* = 0.23, fathers *p* = 0.97).

Several background factors affected the mothers´ experiences of the quality of FCC. Mothers with lower levels of education were more satisfied with the overall quality of FCC (*p* = 0.04), individualized parent education (*p* = 0.04) and information provision (*p* = 0.02) compared to the mothers with higher levels of education. The mothers of twins reported receiving more individualized information than did mothers of singletons (*p* = 0.02). Mothers’ participation in medical rounds increased with infants´ higher gestational age (*p* = 0.03).

## Discussion

This single-center study showed that the architectural change from shared patient rooms to SFR-NICU facilitated parents’ presence significantly but did not increase SSC when the baseline was 3 h per day. The quality of FCC did not increase either, as the level of FCC was already high before the change.

Only a few studies have investigated the effect of architectural change of the NICU on parents’ presence in the unit. Lester at al. [21] showed that mothers were more involved in infant care in the SFR unit than in the open unit, but the duration of mothers’ presence was not measured, and fathers’ participation was not studied. Feeley et al. [22] compared mothers’ presence in an open unit, in a combined pod and in an SFR design and found that mothers spent 44 h per week in the open unit compared to 84 h per week in the SFR unit. Raiskila et al. [23] showed that the duration of parents’ presence was significantly longer in units that provided parents an opportunity to stay overnight: a mean of 19.7 h per day in units with overnight accommodations versus a mean of 5.5 h in units without. When comparing different units, architecture and care culture cannot be separated. In our study, the duration of parents’ presence increased with an architectural change within the same care culture. The duration increased similarly during daytime and nighttime suggesting that SFR unit provides also other benefits than just sleeping facilities.

Interestingly, increased parents’ presence in the SFR-NICU did not translate into increased SSC. A retrospective study in the same hospital [24] showed that the number of SSC episodes quadrupled from 2001 to 2012, before the architectural change, and reached the median among 11 European NICUs in 2013 [23]. This increase before the move might have decreased pressure for the staff to facilitate SSC even further. Despite the large body of literature showing the benefits of SSC [25] what duration of SSC is sufficient to reach these benefits has not been determined. Instead of providing SSC, parents might have used their time interacting with their infant in other ways and participating in caretaking and feeding. Other parental caretaking activities are likely to provide benefits as well. For example, maternal involvement in caretaking has been shown to improve language development in preterm infants [21]. We are not aware of previous studies on the effect of architectural change on SSC. Nevertheless, the parents have been observed to provide more SSC in SFR-NICUs compared to units without SFRs. Raiskila et al. [23] showed a mean of 4.0 h of SSC per day in units that provided parents accommodations to stay overnight versus 1.7 h in units without overnight accommodations.

The care culture is likely to be different in studies comparing units with different architectures, which explains the discrepancy to our findings. Families in our study were actively encouraged to provide SSC in both NICU environments. Pineda et al. [10] did not find a difference in the amount of SSC based on whether the infant was assigned to an open-bay area or to a single-patient room. It seems the practice of actively encouraging parents to provide SSC has more effect on the amount of SSC than does the room type and its privacy. It is also noteworthy that our study unit with shared patient rooms already provided more privacy and better opportunities for parent–infant closeness than a traditional open layout, since the rooms were for two to four patients and resting chairs were provided for parents.

In our study, parents reported the same level of FCC in both NICU environments. We assume that the unit had already reached a family centered mindset in the environment with shared patient rooms through an educational intervention, the Close Collaboration with Parents training, which has been shown to facilitate the FCC culture in the unit and increase the mutual partnership between the staff and parents [26]. The shared patient rooms in the unit also provided a more private environment and better facilities for the parents to participate than is seen in most traditional open layouts. Therefore, the DigiFCC–P suffered from a ceiling effect and was unable to demonstrate potential improvements.

For both NICU architectures, the lowest scores for FCC were reported in emotional support and participation in medical rounds, consistent with earlier studies [27]. In a Norwegian study comparing an open unit and an SFR unit, these elements of FCC were rated at higher levels in the SFR environment [28]. In our study, parents did not report higher scores on emotional support in the SFR-NICU, which was surprising as parent–nurse interaction increased significantly in the study center after the architectural change [14]. Longer interaction episodes should enable the staff to better provide emotional support for the parents. It is essential to understand how to improve emotional support for parents, especially fathers, which seems to have remained suboptimal despite staff education and SFR architecture. In addition, more work is needed to integrate parents into medical rounds, which may be the most challenging part of FCC [17].

Some background factors affected both parents’ presence and their ratings of FCC. We found that the duration of parents’ presence in the unit decreased when there were siblings at home. This is consistent with previous literature [29–31]. However, a recent study reported that siblings did not affect the duration of parents’ presence [23]. This might be explained by better facilities and the willingness to integrate siblings in modern neonatal care. In our study, parents provided more SSC for infants who were twins, explained by an increase in the amount of father–infant SSC. This contradicts the study by Raiskila et al. [23] which showed less SSC in twins. Therefore, it seems that fathers in our study had the opportunity to make an extra effort to participate in the infants’ care with hospitalized twins. In addition, the neonatal staff might have spent more time with twins as mothers of twins were more satisfied with the level of individualized information.

One important background factor was the infant´s gestational age, which often reflected the infant’s medical condition. Higher gestational age associated with more closeness and higher ratings of FCC. Parents of more mature infants, as compared to those of less mature infants, have previously been observed to be more satisfied with care [32] and to have more favorable perceptions of the support from nurses [33]. Parents’ higher education associated with lower ratings of FCC. Previous studies have also reported that higher education associated with lower satisfaction with doctors’ performance, guidance provided to parents [34] and the overall level of NICU care [32] but also with more positive experiences on emotional support [34]. More highly educated parents might have higher expectations on the quality of care in the NICU. The staff needs to be sensitive and observe the individual needs and preferences of each parent in order to provide comprehensive care that fulfills their needs.

As the comparison was done in the same unit at two separate time points, confounding factors were controlled better: the unit had the same care culture and policies regarding care practices, and the staff was mostly the same. The number of admissions was slightly lower in the SFR-NICU and the number of staff was increased by one person. However, the monthly weighted workload remained at the same level in both study points. All eligible families were approached, and the participation rate was high, so there was no significant selection bias. We acknowledge that there are limitations in this study. The number of participating families was rather small. In addition, there were only nine families with extremely preterm (<28 weeks of gestation) infants, so the study results should be generalized with caution to the families of extremely premature infants.

The DigiFCC–P suffered from a ceiling effect, which might be due to the high baseline level of FCC in the study center. It is important to develop the psychometric properties of the existing tools to measure the quality of FCC in neonatal units. The diary method was validated against DigiFCC–P regarding parents’ presence and against nurses’ charting regarding SSC [20]. If there was a reporting bias, it is likely to be an underestimation of presence and SSC as the diary method required active recording. Automated tools to collect data on parent–infant closeness would be valuable in future research and quality improvement.

In conclusion, moving to the SFR-NICU increased parents’ presence, but did not increase parent–infant SSC or parents’ satisfaction with the quality of FCC. The increase from the perspective of the preterm infant was significant as the duration of parent–infant closeness almost doubled. Future research should explore ways to improve emotional support and parent participation in medical rounds.

## References

[1] Winner-Stoltz R, Lengerich A, Hench AJ, OʼMalley J, Kjelland K, Teal M (2018). Staff nurse perceptions of open-pod and single family room NICU designs on work environment and patient care. Adv Neonatal Care.

[2] Harris DD, Shepley MM, White RD, Kolberg KJS, Harrell JW (2006). The impact of single family room design on patients and caregivers: executive summary. J Perinatol.

[3] Fegran L, Helseth S (2009). The parent-nurse relationship in the neonatal intensive care unit context–closeness and emotional involvement. Scand J Caring Sci.

[4] Domanico R, Davis DK, Coleman F, Davis BO (2011). Documenting the NICU design dilemma: comparative patient progress in open-ward and single family room units. J Perinatol.

[5] Jones L, Taylor T, Watson B, Fenwick J, Dordic T (2015). Negotiating care in the special care nursery: parents’ and nurses’ perceptions of nurse-parent communication. J Pediatr Nurs.

[6] van Veenendaal NR, van Kempen, Anne AMW, Franck LS (2019). Hospitalising preterm infants in single family rooms versus open bay units: a systematic review and meta-analysis of impact on parents. Lancet Child Adolesc Health.

[7] Lehtonen L, Lee SK, Kusuda S, Lui K, Norman M, Bassler D (2020). International Network for Evaluating Outcomes of Neonates (iNeo). Family Rooms in NICUs and Neonatal Outcomes: An International Survey and Linked Cohort Study. J Pediatr.

[8] Vohr B, McGowan E, McKinley L, Tucker R, Keszler L, Alksninis B (2017). Differential effects of the single-family room neonatal intensive care unit on 18- to 24-month bayley scores of preterm infants. J Pediatr.

[9] Beck SA, Weis J, Greisen G, Andersen M, Zoffmann V (2009). Room for family-centered care—a qualitative evaluation of a neonatal intensive care unit remodeling project. J Neonatal Nurs.

[10] Pineda RG, Stransky KE, Rogers C, Duncan MH, Smith GC, Neil J (2012). The single-patient room in the NICU: maternal and family effects. J Perinatol.

[11] Reynolds LC, Duncan MM, Smith GC, Mathur A, Neil J, Inder T (2013). Parental presence and holding in the neonatal intensive care unit and associations with early neurobehavior. J Perinatol.

[12] Montirosso R, Del Prete A, Bellù R, Tronick E, Borgatti R (2012). Neonatal Adequate Care for Quality of Life (NEO-ACQUA) Study Group. Level of NICU quality of developmental care and neurobehavioral performance in very preterm infants. Pediatrics.

[13] Lester BM, Hawes K, Abar B, Sullivan M, Miller, Bigsby R (2014). Single-family room care and neurobehavioral and medical outcomes in preterm infants. Pediatrics..

[14] Toivonen M, Lehtonen L, Löyttyniemi E, Axelin A (2017). Effects of single-family rooms on nurse-parent and nurse-infant interaction in neonatal intensive care unit. Early Hum Dev.

[15] Jones L, Peters K, Rowe J, Sheeran N (2016). The influence of neonatal nursery design on mothers’ interactions in the nursery. J Pediatr Nurs.

[16] Liu LX, Mozafarinia M, Axelin A, Feeley N (2019). Parents’ experiences of support in NICU single-family rooms. Neonatal Netw.

[17] Aija A, Toome L, Axelin A, Raiskila S, Lehtonen L (2019). Parents’ presence and participation in medical rounds in 11 European neonatal units. Early Hum Dev.

[18] Saunders RP, Abraham MR, Crosby MJ, Thomas K, Edwards WH (2003). Evaluation and development of potentially better practices for improving family-centered care in neonatal intensive care units. Pediatrics..

[19] Ahlqvist-Björkroth S, Boukydis Z, Axelin AM, Lehtonen L (2017). Close collaboration with parents™ intervention to improve parents’ psychological well-being and child development: Description of the intervention and study protocol. Behav Brain Res..

[20] Axelin A, Raiskila S, Lehtonen L (2020). The development of data collection tools to measure parent-infant closeness and family-centered care in NICUs. Worldviews Evid Based Nurs.

[21] Lester BM, Salisbury AL, Hawes K, Dansereau LM, Bigsby R, Laptook A (2016). 18-month follow-up of infants cared for in a single-family room neonatal intensive care unit. J Pediatr.

[22] Feeley N, Robins S, Genest C, Stremler R, Zelkowitz P, Charbonneau L (2020). A comparative study of mothers of infants hospitalized in an open ward neonatal intensive care unit and a combined pod and single-family room design. BMC Pediatr.

[23] Raiskila S, Axelin A, Toome L, Caballero S, Tandberg BS, Montirosso R (2017). Parents’ presence and parent-infant closeness in 11 neonatal intensive care units in six european countries vary between and within the countries. Acta Paediatr.

[24] Raiskila S, Axelin A, Rapeli S, Vasko I, Lehtonen L (2014). Trends in care practices reflecting parental involvement in neonatal care. Early Hum Dev.

[25] Boundy EO, Dastjerdi R, Spiegelman D, Fawzi WW, Missmer SA, Lieberman E, et al. Kangaroo mother care and neonatal outcomes: a meta-analysis. Pediatrics. 2016;137:e2015223. 10.1542/peds.2015-2238 PMC4702019 26702029

[26] Toivonen M, Lehtonen L, Löyttyniemi E, Ahlqvist-Björkroth S, Axelin A (2020). Close collaboration with parents intervention improves family-centered care in different neonatal unit contexts: a pre-post study. Pediatr Res.

[27] Raiskila S, Lehtonen L, Tandberg BS, Normann E, Ewald U, Caballero S (2016). Parent and nurse perceptions on the quality of family-centred care in 11 European NICUs. Aust Crit Care.

[28] Tandberg BS, Frøslie KF, Flacking R, Grundt H, Lehtonen L, Moen A (2018). Parent-infant closeness, parents’ participation, and nursing support in single-family room and open bay NICUs. J Perinat Neonatal Nurs.

[29] Franck LS, Spencer C (2003). Parent visiting and participation in infant caregiving activities in a neonatal unit. Birth.

[30] Latva R, Lehtonen L, Salmelin RK, Tamminen T (2004). Visiting less than every day: a marker for later behavioral problems in finnish preterm infants. Arch Pediatr Adolesc Med.

[31] Garten L, Maass E, Schmalisch G, Bührer C (2011). O father, where art thou? parental NICU visiting patterns during the first 28 days of life of very low-birth-weight infants. J Perinat Neonatal Nurs.

[32] Hagen IH, Iversen VC, Nesset E, Orner R, Svindseth MF (2019). Parental satisfaction with neonatal intensive care units: a quantitative cross-sectional study. BMC Health Serv Res.

[33] Franck LS, Axelin A (2013). Differences in parents’, nurses’ and physicians’ views of NICU parent support. Acta Paediatr.

[34] Lilo E, Shaw R, Corcoran J, Storfer-Isser A, Horwitz S (2016). Does she think she’s supported? maternal perceptions of their experiences in the neonatal intensive care unit. Patient Exp J..

